# Community pharmacists' decision-making patterns in clinical prescription checking: A simulation-based study

**DOI:** 10.1016/j.rcsop.2025.100569

**Published:** 2025-01-21

**Authors:** Ali Elgebli, Jason Hall, Denham L. Phipps

**Affiliations:** Division of Pharmacy & Optometry, Faculty of Biology, Medicine & Health, The University of Manchester, Stopford Building, Room 1.183, Oxford Road, Manchester M13 9PT, United Kingdom

**Keywords:** Community pharmacists, Clinical checking, Decision-making

## Abstract

**Background:**

Community pharmacists (CPs) make a significant number of decisions on the clinical appropriateness of prescriptions daily to ensure safe and effective use of medications, in a process known as “clinical checking”. The process is complex and is affected by multiple factors in practice. This study aimed to investigate the cognitive processes involved in clinical prescription checking by CPs.

**Method:**

This qualitative study employed a purposive sampling technique to recruit a diverse sample from the population of CPs in England. Engaging in Zoom interviews, participants clinically checked three simulated prescriptions, providing concurrent verbal accounts of their thoughts. The participants' commentaries during the task were audio-recorded, transcribed verbatim, and underwent deductive thematic analysis based on Klein's recognition-primed decision-making (RPD) model.

**Results:**

Twelve CPs from diverse backgrounds and varied working conditions were recruited and completed the online checking task. Making decisions on the clinical appropriateness of prescriptions appeared to be a multi-staged procedure whereby several levels of concerns exist, and pharmacists vary in their ability to recognise and resolve those concerns. CPs behaved in a manner similar to that described by the RPD model; they mostly engaged in pattern-recognition during clinical checking, but adopted a more analytical approach when they recognised an atypical situation. Participants showed more consistency when processing cues and expectancies; however, their subsequent actions exhibited substantial variability, coupled with a degree of hesitancy.

**Conclusion:**

Clinical checking of prescriptions is a multifaceted process in which pharmacists employ a blend of pattern recognition and analytical thinking when making decisions. The process differs notably among pharmacists, underscoring the need to understand the factors driving these variations and any hesitancy in decision- making, as well as their potential impact on patient safety.

## 1 Introduction

In many countries, the primary healthcare sector is responsible for a large proportion of the medication supply to health service users.^1^ As errors in medication use are a significant cause of iatrogenic patient harm,^2^, ^3^, ^4^ there is a need for interventions to minimise their occurrence. A particular concern is prescribing errors (that is, errors in the writing of prescriptions or subsequent monitoring of patients in prescribed medication), which have been estimated to occur at a rate of 5 % in English primary care.^5^ Community pharmacists (CPs) play a key role in minimising the harm that results from primary care prescribing errors,^6^ by performing a “clinical check” of each prescription to confirm that the prescribed medication is suitable for the patient before proceeding to dispense it. This check is typically undertaken in a highly pressurised environment where both pace and decision effectiveness are crucial.^7^^,^^8^ Therefore, it is important to understand how CPs decide on the clinical appropriateness of prescriptions in order to ensure that this part of their task is carried out effectively.

The field of decision-making encompasses diverse theories exploring how individuals arrive at choices, from traditional models emphasising rationality to those considering biases and heuristics in decision outcomes. Influential work by Amos Tversky and Daniel Kahneman^9^ revealed the impact of unconscious biases and introduced the concept of two cognitive systems: one intuitive and quick, the other analytical and deliberate.^10^ This work paved the way for Naturalistic Decision Making (NDM) research, observing decision-making outside controlled environments, notably among professionals like firefighters, pilots, therapists, and military personnel.^11^ NDM extends to healthcare settings, with scenarios featuring ill-structured problems, time constraints, dynamic environments, high stakes, and considerations of organisational goals and multiple stakeholders. For example, NDM research has been applied to study emergency room physicians' decision processes. Studies^12^^,^^13^ show that these physicians often rely on pattern recognition to quickly diagnose and treat acute cases, using rapid mental simulations based on previous experiences rather than methodical, step-by-step analysis, especially under severe time constraints. This approach allows them to make immediate and often life-saving decisions in highly uncertain and dynamic environments.

Decision-making, as framed by the dual-process theory,^10^^,^^14^ operates through two distinct systems: system 1, recognised as pattern-recognition, and system 2, known as analytical thinking. System 1, the domain of pattern-recognition, swiftly generates decisions drawing from past experiences, requiring minimal conscious effort. Conversely, system 2 embodies analytical reasoning, a deliberate, slower approach founded on rational judgment and a methodical search for additional information from prior knowledge and experiences. This multifaceted process plays a fundamental role in the practice of healthcare professionals.^14^^,^^15^

The studies,^16^^,^^17^ have investigated CPs clinical reasoning and diagnostic skills in response to over-the-counter (OTC) queries. Those studies claimed that CPs placed more emphasis on product-selection than on a comprehensive assessment of patients. CPs were found to rely heavily on the use of mnemonics to gather information but demonstrated a lack of comprehension in discerning the nature of inquiries to make with patients. Also, in other work,^18^ it was suggested that CPs oftentimes refer patients to other HCPs to avoid responsibility in making decisions. Though in a recent study^19^ CPs followed an analytical approach and demonstrated confidence in making diagnosis and providing treatment on a simple OTC-encounter (conjunctivitis case).

Furthermore, there is a lack of research focusing on pharmacists' prescription-related clinical decisions, with studies in this area showing inconsistent findings. Croft et al. (2017) identified a systematic approach among CPs, emphasising methodical cue gathering and processing in their clinical decisions. In contrast, Nusair & Guirguis (2017) discovered a tendency among CPs to overlook cues, relying on assumptions and resulting in inadequate information for effective clinical checks. Moreover, recent studies in English community pharmacists revealed that CPs tend to rely on pattern-recognition over analytical methods during prescription checks in practice, a significant oversight in prior research. Additionally, the study by Elgebli et al.^20^ revealed instances where pharmacists deviated from standardised protocols during these checks.

This study employed the “recognition-primed decision (RPD)” model,^11^ to describe CPs decision-making process during simulated clinical checking. The RPD model, a cornerstone within the NDM framework developed by Gary Klein and his colleagues^11^ in the late 1980s, has garnered attention within the healthcare research.^21^^,^^22^ Crucial within dynamic and high-stress environments like pharmacy practice, the RPD model underscores the significance of experience and intuition in swift decision-making. At the heart of the RPD model lies the concept of pattern recognition and intuitive judgment. It suggests that experienced professionals can swiftly make decisions by identifying patterns in current situations that resemble past experiences. This process involves recognising cues and correlating them with similar situations encountered previously, thereby facilitating the identification of a course of action with remarkable speed. Following this recognition, the decision-maker engages in mental simulation, where the chosen action is mentally played out to anticipate its outcomes. This step is instrumental in assessing the action's feasibility and potential success without the need for extensive analytical deliberation.^23^^,^^24^

It is important to note that relying solely on pattern recognition in healthcare can be problematic, particularly in complex cases where unique or subtle variations in patient conditions may not fit recognised patterns, potentially leading to cognitive biases such as anchoring or confirmation bias.^13^^,^^25^ While the RPD model values quick, intuitive judgments, it also highlights the necessity of analytical thinking in complex situations.^25^ Analytical thinking allows clinicians to integrate new, atypical information, ensuring comprehensive evaluations and mitigating risks associated with intuitive decision-making. Given that English community pharmacists have reported relying on pattern-recognition during prescription checking,^26^ the RPD model becomes instrumental in elucidating the process of clinical checking. Hence, we applied it in this study which aimed to investigate the cognitive process involved in clinical prescription checking by CPs.

## 2 Method

### 2.1 Design and materials

This study was qualitative in nature and comprised a low-fidelity simulation of clinical checking combined with concurrent think-aloud technique.^27^, ^28^, ^29^ The task comprised three virtual prescriptions (Refer to Table 1 and supplementary materials): one clinically appropriate prescription (VP-2) and two others, each presenting a specific issue necessitating pharmacist intervention (VP-1 and VP-3). The prescriptions were carefully designed to include medications with varying levels of risk, informed by a comprehensive literature review that identified medicines associated with differing rates of complications and hospitalisations. In the first scenario (VP-1), pharmacists were expected to contact the prescriber to recommend adding a gastroprotective agent, while in the third scenario (VP-3), they were to identify an inappropriate amoxicillin formulation and an incorrect inhaler device. Each scenario was enriched with detailed patient histories and multiple medications to simulate real-world complexity, enabling an understanding of pharmacists' cognitive processes across cases that required varying levels of intervention or no intervention at all.

**Table 1:** Description of the virtual prescriptions presented to participants.

**Prescription**	**Description**
Prescription 1	This prescription was issued for a 65-year-old male, who has been prescribed Naproxen 500 mg (ONE table BD) and Paracetamol 500 (ONE or TWO tabs QDS). His previous medications dispensed (PMR), Included citalopram 10 mg (OD), barrier cream, Hypromellose 0.3 % eye drops, metformin 500 mg (ONE-tab QDS), sitagliptin (OD)
Prescription 2	This prescription was issued to a 39-year-old female, who has been prescribed labetalol 200 mg (ONE table BD), and aspirin 75 mg (TWO tables OD). Her PMR included folic acid 400μg (OD), paracetamol 500 mg (ONE-tab QDS PRN), sertraline 50 mg (OD).
Prescription 3	This prescription was issued to a 5-year-old child, who has been prescribed amoxicillin 500 mg (ONE cap TDS), soluble prednisolone 5 mg (Four tables (20 mg) MANE for 3 days) and aero-chamber plus with mask (1 device). The PMR included Clenil Modulite 50μg/dose inhaler (TWO puffs BD), diprobase cream, salbutamol 100μg/dose inhaler (ONE puff QDS PRN)

The patient scenarios also encompassed diverse demographic groups—paediatric, geriatric, and pregnant patients—with relevant medical histories tailored to each case. To confirm validity, a clinical pharmacist and a panel of community pharmacists reviewed these prescriptions, ensuring their effectiveness in providing insights into the prescription-checking process.

### 2.2 Recruitment and participants

A purposive sampling technique was used to recruit a diverse group of CPs, aiming to include a balanced mix of newly qualified, experienced, contracted and locum professionals. Potential participants were recruited through a combination of social media posts, professional networks of CPs, and snowball sampling techniques. Individuals were provided with an information sheet that detailed their role in the study and the expectations thereof. Participants were informed that the study would involve a clinical checking exercise, during which they would be asked to review three prescription cases. Recruitment continued until data saturation was achieved. The study was granted ethical approval by the University of Manchester Research Ethics Committee (Reference 2021–13,400).

### 2.3 Procedure

Participants undertook an online interview over Zoom, where they worked through the prescriptions while giving concurrent accounts of their thoughts. The verbal reports of participants' work out were audio-recorded and transcribed verbatim by a university-approved transcriber. The interview allowed for prompts and questions to be asked when needed to elicit a full understanding of pharmacists' decisions. Also, participants engaged in a short post-task discussion to reflect on the interview and, in certain occasions, expand on responses of interest.

### 2.4 Analysis

The interview transcripts were subjected to deductive thematic analysis,^30^ using the RPD model as a framework.^11^ This involved systematically aligning interview content with the components of the RPD model: Goals, Cues, Expectancies, and Actions. To ensure validity, the RPD components were rigidly pre-defined: ***Goals*** referred to the ultimate objectives established by pharmacists for each prescription, typically after an initial assessment; ***Cues*** refer to the observable clues in the prescription that inform pharmacists' decision-making, such as the prescribed medications, their dosages, and the patient's list of repeat medications; ***Expectancies*** refer to pharmacists' interpretation of cues and the elements they anticipate as a result. For example, if an NSAID is prescribed to an elderly patient, pharmacists may interpret this as a heightened risk of gastric bleeding and expect a gastroprotective agent to be included; and ***Actions*** are the steps taken following prescription evaluation, such as contacting the prescriber to suggest an alternative formulation or adjusting the medication to meet the patient's needs. Additionally, any text that did not align with the predefined criteria was subjected to thematic analysis and presented as a distinct theme in the results.

AE, a practicing community pharmacist and researcher, led the primary analysis of the study. To minimise potential biases and assumptions inherent to AE's background, he was accompanied by DP, a psychologist, during the first four interviews. This collaboration ensured that AE's personal assumptions did not unduly influence the interview outcomes. Reflective sessions and peer discussions were conducted after each of these initial interviews to further mitigate self-bias and prevent AE's personal experiences from skewing the data. Additionally, the analysis of the interview transcripts was rigorously reviewed in a collaborative effort involving DP, who specialises in ergonomics and human factors within patient safety research, and JH, an academic pharmacist who brings hands-on experience in community pharmacy practice. Consensus was achieved without any disagreements on categorisation. The entire research team had access to the interview transcripts and actively participated in the analytical process, ensuring comprehensive validation and refinement. NVivo software, Version 12 (QSR International, 2017), was used to facilitate the data analysis.

## 3 Findings

### 3.1 Demographic results

The checking task formed a part of longer interviews investigating clinical checking, which lasted, on average, approximately 40 mins. The study included 12 CPs from various organisations, with a gender distribution of 58.3 % male (*n* = 7) and 41.7 % female (*n* = 5). Participants' years of experience in community pharmacy varied, with the majority having 1–3 years (58.3 %, n = 7), followed by 4–6 years (25 %, *n* = 3), 7–10 years (8.3 %, *n* = 1), and over 10 years (8.3 %, n = 1). In terms of employment type, most pharmacists were employed (66.7 %, *n* = 8), while others worked as locums (25 %, n = 3) or were owners (8.3 %, n = 1). The organisation size classified according to the definitions provided by the Royal Pharmaceutical Society^31^; “large multiple” (> 100 pharmacies); “small multiple” (6–99 pharmacies); and “independent” (1–5 pharmacies) (Table 2).

**Table 2:** Characteristics of the community pharmacists participated in the study.

**Pharmacist**	**Gender**	**Years of experience in community pharmacy**	**Nature of work**	**Organisation**
1	Male	One	Employed	Large Multiple
2	Male	Two	Employed	Independent store
3	Male	Two	Employed	Independent chain
4	Female	Six	Employed – chain lead pharmacist	Independent chain
5	Female	Seven	Locum	N/A
6	Female	Five	Locum	N/A
7	Female	One	Employed	Independent chain
8	Male	Two	Employed	Small Multiple
9	Male	One	Employed	Large multiple
10	Male	Twenty	Owner – store manager	Independent pharmacy store
11	Male	Four	Employed – branch manager	Independent chain
12	Female	Two	Locum	N/A

### 3.2 Pharmacists' decision-making journey

#### 3.2.1 Overview and checking patterns

Participants reported extracting cues from patient information, prescription details, and medication history, then formulated expectations and goals, devising an action plan aimed at ensuring the safety and appropriateness of the prescribed medications. However, variations arose in the depth of cue evaluation, the formulation of expectancies, and subsequent actions among pharmacists, resulting in diverse outcomes for each prescription. Table 3 provides a summary of the participants' overall clinical assessments for each prescription.

**Table 3:** A general overview of participants assessments of each virtual prescription.

**VP-1: A prescription of naproxen and paracetamol**	
Issues identified and proposed actions	**N** of participants
*Identified harm of gastric-bleeding*	**10**
Withhold supply and speak to the prescriber regarding a stomach protectant*	**3**
*Withhold and speak to prescriber and suggesting a stomach protectant **OR** a lower dose (250* *mg)*	**1**
*Supply with advice to minimise risks and safety-netting*	**4**
No protection required if one-off **	**2**
**VP-2: A prescription of labetalol and aspirin**	
Issues identified and proposed actions	**N** of participants
*Identifying patient as potentially pregnant*	**12**
*Supply prescription*	**8**
*Supply post-confirmation of pregnancy with patient*	**4**
*Labelling aspirin dose potentially inappropriate and checking resources*	**5**
Identifying aspirin is prescribed for preventing pre-eclampsia***	**3**
**VP-3: A prescription of amoxicillin, prednisolone, and spacer device**	
Issues identified and proposed actions	**N** of participants
*Identified amoxicillin formulation inappropriate*	**10**
*Contacting doctor to amend formulation*	**7**
*Proposing to speak to patient representative prior to deeming formulation inappropriate*	**2**
*Proposed to speak to patient representative if child is able to swallow **OR** suggesting dispersing capsules content into water for ease of administration*	**1**
*Identifying spacer device inappropriate,*	**3**
*Contacting doctor to amend spacer device*	**3**

*Most commonly recommended protectant by pharmacists is PPIs; **Those participants also provided advice and safety-netting (explaining potential signs of and how to respond to side effects); ***Most pharmacists identified patient is suffering from hypertension (due to labetalol), but there was confusion surrounding the use of aspirin, though many said they encountered such doses of aspirin for pregnant women, but not knowing what for exactly (labelled as use in at risk pregnancies)

Participants appeared to primarily rely on pattern recognition during their clinical decision-making journey, seeking familiar cues that aligned with their past experiences, significantly influencing their subsequent actions:

“I know the dose is right [prednisolone dose of 20mg mane], **I've seen that before for that certain age**, up to 20 milligrams once every morning for three days. That's fine. I know this dose is fine”. [9]

“This [aspirin dose of 150mg od] For preeclampsia, but it is unlicensed use, **but I've seen it before where it is initiated by a prescriber, a** midwife or someone from the hospital. For a pregnant lady, yes … I would make a supply, yes” [7]

Yet, they adopted an analytical approach, particularly when there was a mismatch between their expectations and the prescription, or when the generated action seemed implausible:

“The aspirin is a bit of a strange dose […] **I don't know why but I feel like she's pregnant, based on the labetalol and aspirin […] she's also got folic acid on her repeat. So that makes me, like, 99.9 per cent sure she's pregnant … But I'm just going to double check with the BNF for the aspirin dose … Because I know they normally have it once a day in pregnancy […] Yeah, so it is 150, 75 to 150 once daily from 12 weeks gestational until birth of the baby.** So yeah, that makes me 100 per cent sure she's pregnant. So that's fine the aspirin”. [12]

#### 3.2.2 Cues and expectancies

Pharmacists demonstrated a uniform approach to gathering cues, starting with patients' characteristics, and moving down to the prescribed medications and their history (Table 4). Notably, the cues considered relevant includes aspects like age, gender, repeat medications, patient familiarity, prescribed dosage, form, and quantity. These cues formed the foundational elements guiding subsequent decision-making steps.

**Table 4:** A summary of the cues and expectancies processed by CPs for each virtual prescription.

**Situation assessment**		
*Virtual prescription 1:*		
**Cues**	**Expectancies**	**Examples**
“elderly” “naproxen” “History of citalopram” “History of anti-diabetic medicines”	Potential risk of GI bleeding Potential renal issues with anti-inflammatory Prescribing a stomach protecting agent	*“Ideally, I would like omeprazole or a PPI with the naproxen because he's over 65 and so there's increased risk of bleeding and he's on citalopram” [participant 12].* *“First question that comes to me that raises my concern is the naproxen, taking them together with citalopram, because that could be a problem with internal bleeding. And it's for a month. And there's no PPI being prescribed [participant 5]”*
*Virtual prescription 2*		
**Cues**	**Expectancies**	
“female” “childbearing age” “aspirin 150 mg” “labetalol” “history of folic acid” “History of sertraline”	Pregnancy Prevention of pre-eclampsia Aspirin dose range Potential risk of GI complications	*“I feel like she's pregnant, based on the labetalol and aspirin. So, I would possibly…oh, she's also got folic acid on her repeat. So that makes me, like, 99.9 % sure she's pregnant […] The aspirin is a bit of a strange dose. So, I would definitely look that up on the BNF” [participant 12]* *“Labetalol obviously for pregnancy hypertension, and Aspirin can be…also be given in pregnancy. I've had a lot of patients that takes Aspirin in pregnancy” [10]*
*Virtual prescription 3*		
**Cues**	**Expectancies**	
“Paediatric” “amoxicillin capsules” “soluble prednisolone” “History of salbutamol inhaler” “history of steroid inhaler”	Treatment for chest infection Asthma-induced exacerbation Liquid form of amoxicillin Prednisolone dose range Amoxicillin dose range	*“500* *mg Amoxicillin, you've got prednisolone. That seems quite high 20 mg. So, I'm assuming he's had an infection or an exacerbation of his asthma because I can see he's got Clenil and salbutamol. But I feel like his doses are really high, so I would check them on the BNF” [11]* *“I think with anybody under the age of 12, I would definitely be checking doses for antibiotics .... Thinking about formulations as well, capsule, would that be appropriate for a five-year-old, would the liquid be more appropriate? I think it probably would” [participant 4]*

The systematic approach to examining cues was intricately linked to the formation of expectancies in the participants' decision-making process. Expectancies spanned various aspects such as patients' diagnosis, dosage, formulation, potential risks, and protective measures. This iterative process of cue examination and expectancy formulation appeared to be a standardised practice among participants in identifying issues associated with prescriptions' (Table 4). Yet, challenges emerged when participants attempted to translate their interpretations into appropriate actions (as you will see below), pointing towards potential complexities in decision implementation.

It is of significance to note that, in general, participants exhibited a similar approach in processing cues and expectancies. This uniformity was reflected in the majority correctly identifying and addressing the principal issues associated with each scenario (refer to Table 3). However, nuances in the depth of cue assessment and expectancies surfaced, resulting in a select few participants discerning additional concerns within each prescription.

#### 3.2.3 Goals and actions

The overarching goals reported by participants were ensuring the *safety* of prescribed medicines and ensuring the appropriateness of the *formulation*. In some cases, participants judged a medication as being appropriate for the patient solely because the medication fell within an apparent “dose safety range”. This observation highlights a tendency among some participants to prioritise ensuring the safety of prescribed medications while potentially allocating less emphasis on evaluating their effectiveness and indication.

“Normally Prednisolone, from what I know they can give up to 60 milligrams even in a child. so, I'm not too concerned about that”. [7]

“The Aspirin means also the risk during pregnancy. So, I know some patients who are pregnant taking two Aspirin a day. I would actually…yeah, I can…I think this is safe enough to give to the patient. So, if the patient's collecting it that will be fine”. [1]

Furthermore, the differences in cue depth and interpretation, as previously mentioned, reflected the goals set by CPs, categorising them into two clear types: primary goals and secondary goals. *Primary goals* encompass the predominant issues identified by a majority of CPs within each prescription.

o Addressing potential gastric bleeding due to Naproxen for a 65-year-old patient on citalopram.
o Evaluating a potentially high aspirin dose.
o Assessing the appropriateness of the amoxicillin formulation.

On the other hand, *secondary goals* represented the less apparent issues identified by those recognising a broader range of relevant cues compared to their counterparts. These sub-goals involved potential renal threats (VP-1), evaluating sertraline's appropriateness in pregnancy (VP-2), recognising potential gastric issues with 150 mg of aspirin (VP-2), and assessing the accuracy of the inhaler device (VP-3). (See Table 6 for the number of participants who made each observation).

Moreover, participants' actions displayed notable variation despite their recognition of the associated risks in each scenario (refer to Table 5 for specific examples). While a degree of variation was evident across every stage of the decision-making journey, the variability in actions was far more pronounced. Particularly intriguing was the variability in actions, even concerning primary goals where the potential issue was correctly identified.

**Table 5:** Examples of participants variations when making actions.

**Issue identified**	**Variable actions**	**Examples**
*Risk of Gastric-bleeding*	Stop the prescriptions, and ask for enteric coated and PPI to be prescribed	*“I would call the surgery […] for them to prescribe enteric coated ones together with a PPI for a month, and then see where we can go from there” [5]*
	Check the length of time naproxen prescribed for, then advice accordingly	*“I'll put a note to say you're going to take this for quite a long time, the Naproxen. If it is I would recommend he tries to speak to the doctor and get him prescribed with a PPI” [4]*
	Supply prescription with safety-netting	*“I would issue the supply, but I would also let the patient know that there is increased risk of bleeding, so just watch out for any say stomach bleeding or just any… If you see any blood or doesn't feel comfortable, then ring the pharmacy or contact your GP” [7]*
*Inappropriate amoxicillin formulation*	Amend amoxicillin formulation	*“I would ring the GP and say can you change it to solutions, it would be easier for the child” [3]*
	Check with parent/carer if able to swallow or advice dispersing tablet	*“I would check with the parent if the patient were able to swallow. If the patient is not able to swallow, then I could advise the patient to open up the capsule and disperse the insides of the capsule, like mix it with water and give it that way”. [5]*

Nonetheless, it is important to note that during the checking exercise, participants were prompted to replicate real-world decision-making, aligning their actions with their practice. Notably, their actions appeared to be driven not only by their interpretations of cues and expectancies but also by considerations of practicality and the severity of the situation. Mental simulation surfaced in such scenarios, where pharmacists enacted a particular action and swiftly assessed its practicality, weighing its pros and cons and what would actually work. For instance:

“In ideal situation I would call the GP [to change amoxicillin capsule to suspension form; scenario 3] but again pressed for time, I'm leaning more towards actually withholding, not the…I can pass the clinical checking but if the parents come, I would ask first before dispensing it, can this kid actually take the capsules or not” [11].

Moreover, some pharmacists recurrently proposed to speak with the patient prior to making a final decision; that is, making a provisional decision, to be finalised after discussion with the patient. This practice of making provisional decisions, pending further discussions with patients, may indicate an inclination towards extra precautions. However, a noteworthy pattern emerged among participants—displaying a hesitancy to definitively settle on a final decision. Participants deferring actions, in some instances, might have been influenced by the simulated exam conditions rather than their true inclination to engage with patients. As in practical settings, environmental constraints could substantially affect their involvement in decision-making alongside patients. Also, there were no consistent clear-cut decisions on what is deemed inappropriate by all CPs, even in situations where we expected a no-supply decision to always be made.

### 3.3 Differential clinical checking practices

Throughout the participants' decision-making journey, variation between the participants was noted (Table 6). For example, in VP-1, three participants recognised the potential risk of naproxen on an elderly patient's kidneys, considering possible renal impairment. Moreover, not all pharmacists assessed the patients' repeat medications concerning newly prescribed drugs. In VP-2, only seven pharmacists re-evaluated the suitability of the patient's repeat medications after assuming pregnancy status. Moreover, another variation was CPs tendency to make a diagnosis based on the prescribed drugs and/or doses, which not only varied between pharmacists themselves, but also from a scenario to another. Interestingly, no definitive correlation was observed between pharmacists' characteristics and their checking behaviour.

**Table 6:** A list of variations observed during the decision-making process.

**Variations**	**Interviewee pharmacist**											
	**1**	**2**	**3**	**4**	**5**	**6**	**7**	**8**	**9**	**10**	**11**	**12**
**VP-1: A prescription of naproxen and paracetamol**												
*Making a diagnosis*	√		√					√				
*Review appropriateness of repeats medicines*		√						√		√		√
*Considering diabetes*		√		√				√	√		√	
*Considering renal function*		√		√				√				
**VP-2: A prescription of labetalol and aspirin**												
*Making a diagnosis*	√			√	√	√	√	√	√	√	√	√
*Reviewing appropriateness of repeat medicines*				√	√	√		√	√	√	√	√
*Recommending gastro-protection*				√						√		
**VP-3: A prescription of amoxicillin, prednisolone, and spacer device**												
*Making a diagnosis*	√		√		√	√	√	√	√		√	√
*Reviewing appropriateness of repeat medicines*	√		√		√			√			√	
*Checking appropriateness of the aero chamber spacer*					√			√		√		

## 4 Discussion

This study provided valuable data on the cognitive process followed by CPs when clinically checking prescriptions. CPs were found to employ a pattern recognition strategy when validating prescriptions, but they became analytical when the prescription did not align with what they expected and/or when faced with a new situation. Those findings contrast with those reported by Mallinder and Maritini,^19^ where CPs were found to mainly employ an analytical approach in OTC-scenario. However, similar to their study, our results also indicated that participants employed a dual approach—combining pattern recognition and analytical—when making clinical decisions. Furthermore, decisions to supply or withhold prescriptions – which required further details from prescribers or patients, suggesting amendments and/or supply with safety-netting were made by recognition of domain-specific patterns. Upon the screening of prescriptions, CPs identify certain patterns from patient details (e.g., age; gender), item details (e.g., dose, form, duration of treatment) and history (e.g., repeat slip), which they relate to past experiences to inform their actions. This indicates that CPs commonly decide in a fashion consistent with the basic form of the RPD model,^24^ in which pharmacists recognise a scenario as typical and choose the easily generated action (e.g., authorising the combination of amoxicillin and prednisolone for exacerbation).

Nonetheless, an analytical approach was also utilised by participants on certain occasions, primarily when the generated action was deemed suboptimal or when expectations were violated. The transition to analytical approach resonates with the RPD model, which, although primarily reliant on pattern recognition, integrates analytical processes for diagnosing and evaluating courses of action (COA). Yet, the model diverges from traditional analytical frameworks by prioritising depth of understanding over breadth and comparison of alternative COAs.^11^ Instead of comparing multiple alternatives, the RPD model focuses on recognising familiar patterns and evaluating a primary option, modifying it as necessary. The participants' analytical tendencies in this study reflected those outlined by the RPD model, which contrasts with traditional analytical models ^32^^,^^33^^,^^34^ that prioritise comparing multiple courses of action simultaneously to find the best one.

In this study, CPs transitioned from pattern recognition to analytical thinking upon encountering specific “triggers” such as atypical doses, risky drug combinations, or conflicting patient histories. For example, in Scenario 1, the patient's history of citalopram use, coupled with a new prescription for naproxen, raised concerns about gastrointestinal bleeding, particularly when the expected inclusion of a protective agent was absent. Similarly, in Scenario 2, a higher-than-anticipated dose of aspirin prompted pharmacists to adopt a more analytical approach, as did the prednisolone dose and amoxicillin formulation in Scenario 3. These cases demonstrate how unusual does, drug combinations or patient histories can prompt pharmacists to reassess their initial responses. As discussed previously, this shift aligns with the RPD model, in which analytical reasoning complements pattern recognition in atypical situations.

The approach by which participants processed cues was consistent with that described by Croft et al.^35^ where pharmacists initially screen cues to generate a hypothesis which inform their decisions. Our participants were also forming expectations (on the basis of the cues) that needed to be addressed for the prescription to be deemed appropriate. These expectations encompassed dosage, formulation, potential risks, and protective measures (Table 4). Furthermore, as in Nusair & Guirguis'^36^ study, CPs tended to frequently make assumptions (expectancies) on the reason-for-use to facilitate decision-making, but such assumptions were made selectively depending on the situation. In other words, CPs tended to guess why the medications were prescribed when the answer was deemed essential (e.g., aspirin for pre-eclampsia helped determining whether dose is appropriate). Interviewees predominantly made correct assumptions about the patients' diagnosis; however, the cognitive task was not designed to test CPs' diagnostic abilities, requiring simple diagnostic decisions. Therefore, while previous research^37^ indicated that CPs lacked necessary diagnostic skills, our findings do not provide much insight into this particular issue.

An interesting finding of this study was pharmacists' *actions*, which were somewhat inconsistent and were accompanied by a degree of hesitancy. A few pharmacists exhibited hesitance to making actions; frequently proposing to speak to the patient before a decision is finalised. Patients can be extremely valuable in fulfilling knowledge gaps about their health,^38^ however, interviewees oftentimes opted to speak to the patient even in situations where the decision was understood to be clear [e.g., amending inappropriate amoxicillin formulation for 5-year's old (VP-3)]. Such hesitance was alluded to in previous research^18^ where CPs were reluctant to make independent decisions to avoid responsibility. Others^39^^,^^40^ described this approach as lack of confidence in clinical knowledge as a barrier to implementing a change in community pharmacies.

Furthermore, the depth in CPs clinical involvement in each prescription varied (Table 3) suggesting that clinical checking is a multi-staged process, whereby several levels of concern exist. The multi-staged checking process ranges from a basic check identifying the obvious issues to an advanced check considering more cues and detecting less obvious problems; interestingly there was no clear link between pharmacists' characteristics and level of checking. This was apparent in VP-3, where almost all pharmacists focussed on verifying appropriateness of amoxicillin (most obvious issue) and prednisolone (high-risk drug), and only three participants identified the inappropriate spacer device. This illuminates findings from previous work^26^ which identified a difference in the depth of how CPs perceive clinical checking, and that pharmacists tended to pay less attention to those deemed low-risk. Though, there still are unwanted consequences for such practice; for example, in this instance, the child not receiving benefit of spacer (ineffective) or discarding spacer and requesting a new one from prescriber (cost implications).

Variation in CPs performance was explained in previous studies examining CPs' response to OTC clinical decisions.^17^^,^^41^ However, the variation discovered in this study remains of interest because, as opposed to OTC recommendations, clinical checking is governed by rigid standard operating procedures (SOPs). Also, previous studies^35^^,^^36^ investigating CPs approach to prescription-related clinical decisions have not established a variation across CPs. Hence, future research should investigate the reasons for variations in clinical checking and the impact of such variations on patients' health. Furthermore, despite the checking task in this study being completed in a quiet, uninterrupted setting, there were incidences of missed *cues*, *expectancies,* and inappropriate *actions*, which may partially answer why we still seeing medication-related harm taking place. Therefore, additional support is needed to enrich CPs decision-making skills; here, we provided a thorough assessment of each variable involved in the checking process, such information should assist in tailoring training programmes and CPDs to CPs needs.

### 4.1 Strengths and limitations

In this work, we sought to recruit pharmacists from a range of backgrounds, in order to represent the different views and experiences that exist among CPs in England. However, the small sample size may have limited the extent of our representation, as participants' were primarily from northern England and the Midlands, and highly experienced pharmacists were underrepresented in the sample. Also, the data collection strategy provided us with a concurrent insight into the participants' cognitive processes. However, it is possible that the act of providing a verbal protocol might have intruded with task-related cognitive processes; also, it is possible that only knowledge that was being actively processed in “working memory” could be articulated, leaving “nonconscious” processing inaccessible.^42^ However, when asked, interviewees said that thinking-aloud had not impacted or altered their thinking.

Another strength is the validation process for the prescriptions used in the study. Prior to data collection, independent clinical pharmacists and a panel of senior pharmacists—drawn from the same eligible participant pool—reviewed the prescriptions to ensure their relevance and accuracy. Additionally, this group reconvened after the results were analysed, providing a form of response validation. Although they did not participate in the main exercise, their review helped confirm the findings and added an extra layer of reliability,

Finally, the use of a simulation design allowed for an experimental control over the task presented to participants, and hence made it feasible to investigate their conduct of a task that occurs extensively in practice. However, this is at the expense of a limited representation of the “pillars of naturalistic decision making” that might affect the task in practice (that is the interaction with patients, time stress, ill-structured problems, and organisational norms),^43^ which limits the transferability of the findings.

## 5 Conclusion

Clinical checking is a complex, multi-staged process whereby several levels of concerns may co-exist, and pharmacists varied in detecting and responding to such concerns. CPs appeared to employ a mix of pattern recognition and analytical thinking with the latter strategy used when pattern recognition is unlikely to produce a safe decision. Overall, pharmacists demonstrated more consistency when processing cues and expectancies, though their actions were variable, and accompanied with a degree of hesitancy.

These findings are of a particular significance to researchers and policy-makers who are keen on improving patient safety in primary care. Inconsistent clinical checking and hesitancy can compromise care quality and potentially undermine the effectiveness of established safety protocols. These issues warrant further investigation, especially given that clinical checking is guided by rigid SOPs to maintain minimum safety standards. Future research should explore how CPs' checking aligns with these protocols, examine the reasons behind any deviations, and identify strategies to support pharmacists in conducting more thorough prescription checks.

## Funding

This work was funded by the Libyan Ministry of Education.

## Declaration of Generative AI and AI-assisted technologies in the writing process'

During the preparation of this work the lead author(s) used Bing AI technology strictly to provide language guidance for the initial draft, which went through rounds of revisions by the authoring team. After using this tool, the author(s) reviewed and edited the content as needed and take(s) full responsibility for the content of the publication.

## CRediT authorship contribution statement

**Ali Elgebli:** Writing – original draft, Methodology, Funding acquisition, Formal analysis, Data curation, Conceptualization. **Jason Hall:** Writing – review & editing, Supervision, Conceptualization. **Denham L. Phipps:** Writing – review & editing, Validation, Supervision, Methodology, Conceptualization.

## Declaration of competing interest

None to declare.

## Data Availability

Data available on request.

## Appendix A Supplementary data

Supplementary material Image 1
